# Editorial: Fibrinolysis in Immunity

**DOI:** 10.3389/fimmu.2020.00582

**Published:** 2020-03-31

**Authors:** Krasimir Kolev, Robert L. Medcalf

**Affiliations:** ^1^Department of Medical Biochemistry, Semmelweis University, Budapest, Hungary; ^2^Australian Centre for Blood Diseases, Monash University, Melbourne, VIC, Australia

**Keywords:** hemostasis, fibrin, contact system of coagulation, plasmin, staphylocoagulase, animal model

Haemostasis and inflammation are tightly interrelated processes involved in the resolution of tissue damage. The formation and dissolution of fibrin meshwork is a key interface of these processes. Recent studies have demonstrated the contribution of specific fibrin structures (fibrin bio-film) in the protection against microbial infection in external blood clots (1, 2). Oppositely, different bacteria have been shown to enhance their pathogenicity through modulation of fibrin formation and lysis and a number of molecular events have been described for this pathogen-host interaction (3). Many studies from the past decade provided a sound support for the concept that neutrophil extracellular traps (NETs, extracellular DNA expelled from neutrophils and decorated with released neutrophil enzymes and other proteins) form a secondary scaffold -in addition to fibrin- that increases the mechanical and lytic stability of arterial and venous thrombi (4). Several new data have identified the contact coagulation pathway (Factor XII (FXII), prekallikrein, high molecular weight kininogen) as a key junction mechanism involved in the coordinated activation of blood clotting, inflammation, complement system, and fibrinolysis (5). In contrast, key components of the fibrinolytic system have been reported to attenuate the innate immunity; e.g., tissue-type plasminogen activator (tPA) suppresses the macrophage response to potent inflammatory stimuli (e.g., lipopolysaccharide) (6).

This Research Topic presents several review and original research articles that summarize some recent advances and reveal new facts improving our understanding of the two-way interplay of innate immunity and fibrinolysis.

Based on continuously expanding experimental evidence, Renné and Stavrou elaborate a convincing concept for the engagement of FXII in the propagation of infections and in the control of immune cell behavior. In disease states of infection, the surface of bacterial pathogens or polyphosphates released by them promote the autoactivation of FXII and its pro-coagulant action contributes to the bacterial invasion as suggested by the decreased bacterial load in functional FXII deficiency. Under conditions of sterile inflammation (wound repair, autoimmunity) FXII recruits neutrophils through activation of cell surface receptors and the released NETs form a link to fibrin, the end-product of its pro-coagulant activity.

On a related theme, Maas proposes an interesting link between the fibrinolytic system, the contact system and bradykinin production where plasmin is proposed to be central to all points of this inflammatory triangle. From a structural viewpoint, FXII and tPA are highly homologous and it has been known for decades that while FXII can activate plasminogen, plasmin can also activate FXII. This author has coined the term “plasminflammation” to encapsulate this interesting relationship between plasmin and inflammation.

The original study reported by Farkas et al. reveals important differences in the structural, biomechanical and lytic properties of fibrin and plasma clots generated by staphylocoagulase from *Staphylococcus aureus* and by the endogenous blood coagulation system. Staphylocoagulase forms more fragile fibrin meshwork with higher porosity than the endogenous fibrin, which is more sensitive to mechanical disintegration by shear forces and to enzymatic digestion by the fibrinolytic system. These structural and functional features of the staphylocoagulase-induced clots provide a mechanistic explanation for the bacterial propagation from valvular vegetations in *Staphylococcus* endocarditis and exemplify how a pathogen takes advantage from the endogenous blood coagulation and fibrinolysis for its maintenance and invasion.

A similar *in vitro* approach was employed in the study reported by Litvinov et al. who addressed the structural and rheological alterations in plasma clots induced by sterile inflammation in patients with systemic lupus erythematosus (SLE). The SLE plasmas formed more rigid fibrin matrix with faster kinetics of formation and these prothrombotic alterations correlated with systemic markers of inflammation (erythrocyte sedimentation rate, levels of fibrinogen, β-2 globulin, antibodies to β-glycoprotein 1, and IgM). The SLE clots showed increased susceptibility to fibrinolysis suggesting a kind of compensatory mechanism that developed in the patients.

The above-mentioned *in vitro* approaches provide valuable mechanistic information, but they miss the effects of viable intact tissues, blood vessels, and natural blood circulation. Animal models (primarily mouse) are a “gold-standard” for preclinical evaluation of inflammation-related thrombosis, because they allow to explore the entire complexity of factors influencing the pathogenesis of thrombosis. The comprehensive review by Beristain-Covarrubias et al. provides a critical appraisal of the utility of such models to understand the motive forces and consequences of infection-driven thrombosis with a special focus on *Salmonella* infection. Based on their own experience and extensive literature data the authors communicate helpful hints how genetic manipulations and pharmacological tools could be applied to identify safe targets in the hemostatic/fibrinolytic system and in the function of platelets and neutrophils in order to influence infection-mediated mechanisms in thrombosis at whole organism level.

The relationship between fibrinolysis and inflammation is addressed in a review article by Mukhopadhyay et al., where these authors have considered this in the context of venous thrombus resolution. Fibrinolysis driven by urokinase appears crucial in the venous clot resolution together with modulation of the inflammatory response. The intriguing relationship between fibrinolysis and immune function is reported by Draxler et al. Using a mouse model of ischemic stroke, the cerebral ischemic event caused the expected immunosuppressive effect, but this was further worsened following administration of mouse tPA. This in turn prompted greater neurological deficiency. This immunosuppressive effect of tPA involved both plasmin dependent and independent mechanisms and also raises the question as to whether tPA thrombolysis poses a greater post-stroke infection risk due to this immunosuppressive effect of tPA.

The field of Fibrinolysis has extended far beyond its classical role in fibrin removal. Arguably the most recent advances have been made in the field of immunology and this has already extended into the clinical arena where the use of anti-fibrinolytic drugs were reported to reduce post-operative infection rates (7). We are confident that researchers interested in the latest developments in fibrinolysis particularly in relation to inflammation and immune function will find the contributions in this special issue of *Frontiers in Immunology* enlightening.

## Author Contributions

KK and RM wrote the manuscript.

### Conflict of Interest

The authors declare that the research was conducted in the absence of any commercial or financial relationships that could be construed as a potential conflict of interest.

## References

[1] Macrae FL Duval C Papareddy P Baker SR Yuldasheva N Kearney KJ. A fibrin biofilm covers blood clots and protects from microbial invasion. J Clin Invest. (2018) 128:3356–68. 10.1172/JCI98734 29723163 PMC6063501

[2] Opneja A Kapoor S Stavrou EX. Contribution of platelets, the coagulation and fibrinolytic systems to cutaneous wound healing. Thromb Res. (2019) 179:56–63. 10.1016/j.thromres.2019.05.001 31078121 PMC6556139

[3] Liesenborghs L Verhamme P Vanassche T. Staphylococcus aureus, master manipulator of the human hemostatic system. J Thromb Haemost. (2018) 16:441–54. 10.1111/jth.13928 29251820

[4] Varjú I Kolev K. Networks that stop the flow: A fresh look at fibrin and neutrophil extracellular traps. Thromb Res. (2019) 182:1–11. 10.1016/j.thromres.2019.08.003 31415922

[5] Maas C Renné T. Coagulation factor XII in thrombosis and inflammation. Blood. (2018) 131:1903–9. 10.1182/blood-2017-04-569111 29483100

[6] Mantuano E Azmoon P Brifault C Banki MA Gilder AS Campana WM. Tissue-type plasminogen activator regulates macrophage activation and innate immunity. Blood. (2017) 130:1364–74. 10.1182/blood-2017-04-780205 28684538 PMC5600142

[7] Draxler DF Yep K Hanafi G Winton A Daglas M Ho H. Tranexamic acid modulates the immune response and reduces postsurgical infection rates. Blood Adv. (2019) 3:1598–609. 10.1182/bloodadvances.2019000092 31126915 PMC6538872

